# The volumes of amygdala subregions and peripheral programmed cell death protein‐1 levels are associated with cognitive decline in individuals with knee osteoarthritis

**DOI:** 10.1002/brb3.70042

**Published:** 2024-09-30

**Authors:** Peiling Zeng, Baoru Zhao, Ming Li, Yajun Wang, Guiyan Cai, Ruilin Chen, Lidian Chen, Jiao Liu

**Affiliations:** ^1^ College of Rehabilitation Medicine Fujian University of Traditional Chinese Medicine Fuzhou Fujian China; ^2^ School of Traditional Chinese Medicine Capital Medical University Beijing China; ^3^ Affiliated Rehabilitation Hospital Fujian University of Traditional Chinese Medicine Fuzhou Fujian China; ^4^ National‐Local Joint Engineering Research Center of Rehabilitation Medicine Technology Fujian University of Traditional Chinese Medicine Fuzhou Fujian China; ^5^ Traditional Chinese Medicine Rehabilitation Research Center of State Administration of Traditional Chinese Medicine Fujian University of Traditional Chinese Medicine Fuzhou Fujian China; ^6^ Laboratory of Orthopedics & Traumatology of Traditional Chinese Medicine and Rehabilitation (Fujian University of Traditional Chinese Medicine) Ministry of Education Fuzhou Fujian China

**Keywords:** amygdala subregions, brain region volume, cognitive decline, knee osteoarthritis, PD‐1 level

## Abstract

**Background:**

Persistent pain is a prominent symptom of knee osteoarthritis (KOA) and has been associated with cognitive decline in individuals with KOA. The amygdala, a complex structure consisting of nine subnuclei, and programmed cell death protein‐1 (PD‐1) levels play crucial roles in pain regulation and cognitive processing. This study aims to investigate the relationships among amygdala subregion volumes, cognitive function, and PD‐1 levels to elucidate the underlying mechanism of cognitive decline in KOA.

**Methods:**

In this cross‐sectional study, we recruited 36 patients with KOA and 25 age/gender‐matched healthy controls for neuropsychological tests, structural magnetic resonance imaging scanning, and measurement of serum PD‐1 levels. We used the atlas provided by FreeSurfer software to automatically segment the amygdala subnuclei. Subsequently, we compared the volumes of amygdala subregions between groups and explored their correlation with clinical scores and PD‐1 levels.

**Results:**

Compared to healthy controls, individuals with KOA exhibited significantly lower scores on global cognition tasks, such as long‐delay free recall, short‐delay free recall, and immediate recall tasks. Moreover, they displayed decreased volumes in lateral nucleus basal nucleus paralaminar nucleus while showing increased volumes in accessory basal nucleus, central nucleus, medial nucleus, and cortical nucleus. Within the KOA group specifically, paralaminar volume was negatively correlated with immediate recall scores; pain scores were negatively correlated with global cognition; basal volume was negatively correlated with PD‐1 levels.

**Conclusion:**

Our findings highlight those alterations in amygdala subregion volumes along with changes in serum PD‐1 levels may contribute to observe cognitive decline among individuals suffering from KOA.

## Graphical abstract

This study indicates that individuals with knee osteoarthritis (KOA) exhibit significantly cognitive impairment compared to healthy individuals, with a negative correlation between pain degree and cognitive performance and structural changes in certain amygdala subfields. Specifically, reduced volumes in these subregions were linked to immediate recall and PD‐1 levels. These findings suggest that alterations in amygdala subregion volumes and serum PD‐1 levels may contribute to the cognitive decline in KOA individuals.

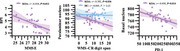

## 1 INTRODUCTION

Chronic pain is a primary symptom of knee osteoarthritis (KOA), which results in a decline in knee function and quality of life (Sharma, 2021), as well as an increased risk of dementia and cognitive impairment (Guo et al., 2022). The results of a recent systematic review comprising retrospective cohort studies indicate a significant correlation between pain and an elevated risk of developing all‐cause dementia (OR = 1.26) as well as Alzheimer's disease (OR = 1.28) (Yuan et al., 2023). The risk of cognitive declines in older adults who experienced persistent pain increased by 21% for every 2‐year interval (Bell et al., 2022). Previous research has indicated that individuals with KOA exhibit global cognitive and memory impairments (Lin, G et al., 2022), with pain intensity showing a negative correlation with global cognition, executive function, and memory function (Crowley et al., 2022). However, the underlying mechanisms remain unclear.

Although the precise mechanisms remain elusive, an increasing body of research indicates the presence of a shared neuroanatomical foundation between the cognitive task processing system and the pain processing system, suggesting a close interconnection and reciprocal influence between these two systems (Moriarty et al., 2011; Xu et al., 2024). The brain regions undergoing structural and functional changes as a consequence of chronic pain not only encompass pain processing but also exert an impact on attention, memory consolidation, and cognitive processing (Innes & Sambamoorthi, 2020). Our recent study utilizing large‐scale cross‐sectional and longitudinal cohorts (*N* = 9,344) revealed an accelerated pattern of brain aging in KOA, primarily driven by hippocampal changes that predict subsequent memory decline and incidents of dementia during a 5‐year follow‐up period. Furthermore, we identified pleiotropy of the SLC39A8 gene related to both brain‐aging acceleration and KOA pathology while observing spatially transcriptional associations with regional contributions to brain‐aging accelerations. These findings underscore a heritable morphological pattern linking accelerated brain aging to cognitive decline as well as increased risk for dementia in individuals with KOA (Zhao L et al., 2024). Therefore, cognitive decline in patients with KOA may be associated with abnormal changes in brain regions.

Brain magnetic resonance imaging (MRI) has revealed abnormal changes in brain structure among patients with KOA (Guo et al., 2021; Mao et al., 2016). However, limited studies have investigated the association between altered brain structure and cognitive decline in individuals with KOA. The amygdala, located in the medial temporal cortex and considered a component of the limbic system (Liao et al., 2021), is traditionally associated with emotional processing (Llorca‐Torralba et al., 2019); however, recent research has highlighted its role in cognitive processing (Yue et al., 2018; Zanchi et al., 2017). A longitudinal MRI study found that Alzheimer's disease patients experienced a 3.79% atrophy rate of the amygdala within 1 year (Fjell et al., 2009). Furthermore, reduced amygdala volume was observed among those experiencing cognitive decline compared to healthy controls (Zanchi et al., 2017), and decreased right amygdala volume showed significant correlation with global cognition among individuals with subjective cognitive decline (Yue et al., 2018). Nevertheless, direct evidence linking amygdala volume and cognitive deficits specifically in KOA individuals remains lacking.

The amygdala is a complex structure comprising nine subnuclei (Saygin et al., 2017), which have been independently associated with other cortical areas (Hirato et al., 2021; Rizvi et al., 1991). The lateral nucleus serves as a crucial center for information input in the amygdala (Moscarello & LeDoux, 2014) and plays a significant role in memory consolidation (Maddox & Schafe, 2011), whereas the central nucleus, as the main output of the amygdala (Wilensky et al., 2006), has been found to be involved in spatial learning and memory (László et al., 2022). Recent research has demonstrated that the accessory nucleus is linked to global cognition (Liu S et al., 2022). These studies suggest that different subnuclei within the amygdala may be responsible for distinct cognitive domains.

The programmed cell death protein 1 (PD‐1) plays a crucial role as an immunosuppressive molecule, exerting regulatory control over the immune system by effectively suppressing the inflammatory activity of T cells. Moreover, it has been implicated in modulating neuroinflammatory processes associated with various central nervous system disorders, including chronic pain and Alzheimer's disease (Zhao J et al., 2021). It has been suggested that PD‐1 contributes to pain symptoms experienced by people with KOA (Montesino‐Goicolea et al., 2022). The protein is also present in neurons within the central nervous system, where it plays a pivotal role in regulating neuronal excitability, synaptic transmission, and plasticity (Jiang et al., 2020). The blockade of the PD‐1 signaling pathway has been increasingly demonstrated in rodent studies to induce an immune response, leading to the recruitment of monocyte‐derived macrophages into the brain. This process facilitates the elimination of pathological amyloid‐β (Aβ) plaques and subsequently enhances cognitive function in mice with Alzheimer's disease (Rosenzweig et al., 2019; Xing et al., 2021). Recent research indicates significantly higher levels of peripheral blood PD‐1 among patients with Alzheimer's disease than healthy individuals do (Wu et al., 2022). These studies suggest that it may serve as a biomarker for predicting cognitive decline. Therefore, investigating the relationship between peripheral PD‐1 levels and brain regions may be beneficial in gaining further insights into the potential mechanisms of cognitive decline in individuals with KOA.

The aim of this study is to investigate the potential mechanism underlying cognitive decline in individuals with KOA by examining the correlation among amygdala subregion volumes, clinical scales, and PD‐1 levels. Our hypothesis posits that changes in amygdala subregion volumes and PD‐1 levels among KOA patients are associated with cognitive impairment.

## 2 METHODS

### 2.1 Study design

A cross‐sectional study was conducted in Fuzhou City, China and registered with the Chinese Clinical Trial Registry (ChiCTR‐IOR‐16009308). This study received approval from the Medical Ethics Committee of the Affiliated Rehabilitation Hospital of Fujian University of Traditional Chinese Medicine, and all procedures were performed in accordance with the Declaration of Helsinki. Prior to participation, informed consent was obtained from all subjects.

### 2.2 Participants

This study recruited 36 elderly patients with KOA (8 males and 28 females) aged between 59 and 66.75, as well as 25 age‐ and gender‐matched healthy controls (8 males and 17 females) aged between 57.50 and 68. The diagnosis of KOA was made by a rheumatologist from the Affiliated Rehabilitation Hospital of Fujian University of Traditional Chinese Medicine based on the criteria established by the American Rheumatism Association (Altman et al., 1986).

The inclusion criteria for the KOA group were as follows: (i) meeting the diagnostic criteria for KOA according to the American College of Rheumatology/Arthritis Foundation (Altman et al., 1986); (ii) attaining a Kellgren–Lawrence grade II or III in X‐ray diagnosis (Holzer et al., 2015); (iii) aged between 40 and 70 years old; (iv) right‐handedness; (v) having a body mass index (BMI) ≤30 kg/m^2^; (vi) referring to our previous study (Liu, Chen, et al., 2019), having an average pain severity score on the brief pain inventory (BPI) > 2 points over the past 24 h; and finally, (vii) scoring above 24 on mini‐mental state examination (MMSE).

The exclusion criteria for the KOA group included individuals who had (i) undergone knee surgery within the past 6 months, including arthroscopic procedures; (ii) received intra‐articular glucocorticoid injections within the last 3 months; (iii) experienced knee pain caused by rheumatic disease or other inflammatory conditions; (iv) demonstrated severe knee deformity leading to an inability to walk independently or reliance on assistive devices; (v) suffered from severe cardiovascular and cerebrovascular diseases, musculoskeletal disorders, or mental illness that impeded voluntary participation in the study; (vi) had contraindications for MRI scans like implanted pacemakers, metal stents/dentures/hearing aids/high fever/claustrophobia/etc.; and (vii) had contraindications for fasting blood samples such as bleeding disorders.

The inclusion criteria for the healthy control (HC) group were as follows: (i) age ranging from 40 to 70 years; (ii) right‐handedness; (iii) BMI≤30 kg/m^2^; (iv) absence of pain disorder with a BPI score of 0; and (v) MMSE score > 24. Exclusion criteria for the HC group included the presence of any neurological or mental illness that could impede cooperation with researchers in completing the study and contraindications for MRI scans.

### 2.3 General demographic and clinical scale assessment

The demographic characteristics of the subjects, including age, gender, BMI, and years of education, were collected. The BPI scale (consisting of nine items) was utilized to assess the average pain intensity experienced by individuals with KOA within the last 24 h. Each item was rated on a scale ranging from 0 to 10, with higher scores indicating greater severity and impact of pain. The Western Ontario and McMaster Universities Osteoarthritis (WOMAC) scale, widely employed for evaluating disease progression or treatment response in patients with KOA, has gained extensive recognition globally following comprehensive consultations with KOA patients, rheumatologists, and epidemiologists (Collins et al., 2011). Previous research has shown that the WOMAC scale has high reliability for pain, stiffness, and physical function at .91, .81, and .84, respectively, and discriminant validity tests indicated that the WOMAC scale better distinguishes between participants with different levels of knee joint issues (Salaffi et al., 2003).The three aspects are assigned respective scores of 20 points, 10 points, and 68 points. Higher scores indicate more severe conditions.

The MMSE was employed to assess the participants' overall cognitive function. Scores on this assessment range from 0 to 30, with lower scores indicating poorer cognitive performance. The Chinese Revision of the Wechsler Memory Scale for adults (WMS‐CR) was utilized to evaluate various aspects of memory, including long‐delay free recall, short‐delay free recall, and immediate recall tasks, such as experience and orientation, mental control A (counting from 1 to 100), mental control B (counting from 100 to 1), and mental control C (repeatedly adding 3 or 4 starting from number one until reaching 49) for long‐delay free recall; picture recall; visual recognition; visual reproduction; associative learning; touch; and comprehension memory for short‐delay free recall. Additionally, the digit span task was administered for immediate recall assessment (Elwood, 1991). All subtest scores (excluding experience and orientation) were aggregated to obtain a composite total score, whereas the memory quotient (MQ) was calculated using an age‐weighted scoring system. Higher scores across all subtests as well as MQ indicate superior memory function.

### 2.4 The measurement of the PD‐1 levels

In accordance with the manufacturer's recommended testing protocols (Yanaba et al., 2016) and building upon our previous research (Liu J et al., 2019), we performed a quantitative assessment of peripheral serum levels of PD‐1 utilizing enzyme‐linked immunosorbent assay (ELISA). Blood samples were collected from 36 individuals diagnosed with KOA, who underwent a 12‐h fasting period prior to blood collection. A trained healthcare professional performed venipuncture on the upper arm using a sterile serum separation tube. The collected blood samples were allowed to coagulate at room temperature for 15 min, followed by centrifugation at 3000 RPM for 3 min to obtain serum, which was promptly stored in a refrigerator at −80°C. Subsequently, quantitative measurements of serum PD‐1 were conducted using an ELISA kit following the manufacturer's instructions (ELISA kits from Huamei Biological Engineering Co., LTD; Cusabio Biotech Cat CSB‐E13643h, RRID:AB_2936220).

### 2.5 Brain MRI acquisition

After collecting general demographic characteristics and clinical scales, all eligible participants underwent a 3.0‐T MRI scan (General Electric,) using an 8‐channel head coil. Participants were instructed to keep their eyes closed and remain still while minimizing movement during the scan; spongy pads held their heads in place and earplugs reduced noise impact. Structural MRI images were acquired with parameters, including a repetition time (TR) of 2100 ms, an echo time (TE) of 30 ms, a voxel size of 3.125 mm × 3.125 mm × 3.6 mm; *T*1‐weighted images had a flip angle of 15° (FA), field of view (FOV) of 240 mm and thickness of 1 mm across 160 slices; conventional *T*2‐weighted images were also obtained to rule out intracranial organic lesions.

### 2.6 The process of brain MRI data

The MRI scan data, exported from the GE Signa workstation (GE Advanced Workstation) in DCOM format, were converted into img format files compatible with SPM12 (Statistical Parametric Mapping 12) software available at https://www.fil.ion.ucl.ac.uk/spm/software/spm12/. Subsequently, each newly exported *T*1/*T*2 images underwent meticulous examination using MRIcron software. Any data that did not meet the specified requirements, such as dislocation or incomplete structure, were excluded from further analysis. Additionally, *T*2‐weighted imaging was thoroughly evaluated by experienced attending‐level doctors in the imaging department to exclude patients with brain tumors or other brain diseases.

The FreeSurfer software version 7.1.0 (http://surfer.nmr.mgh.harvard.edu/, RRID:SCR_001847) utilizes a probabilistic atlas constructed from high‐resolution MRI data to perform automated segmentation of the amygdala nuclei. Prior to amygdala volume segmentation, we standardized all subject images using this software and subsequently employed the FreeSurfer‐provided probabilistic atlas to segment the entire amygdala into nine subregions (Saygin et al., 2017), including the lateral nucleus, basal nucleus, accessory basal nucleus, anterior amygdaloid area, central nucleus, medial nucleus, cortical nucleus, cortico‐amygdaloid transition area, and paralaminar nucleus. For analysis purposes, left and right amygdala subregions were combined.

## 3 STATISTICAL ANALYSIS

All data, including general demographic information, clinical scales, and amygdala subregion volumes, were analyzed using SPSS statistical software (version 25.0; IBM Corp.; https://www.ibm.com/products/spss‐statistics, RRID:SCR_016479). A two‐tailed test with a significance level of *p *< .05 was used to determine statistical significance. The normality of quantitative data was assessed using the Shapiro–Wilk test. Normally distributed quantitative data are presented as mean ± standard deviation and compared between the KOA and HC groups using an independent samples *t*‐test. Non‐normally distributed quantitative data are presented as median (25th‐75th percentile) and compared between groups using the Mann–Whitney *U* test for independent samples. Qualitative data are reported as frequencies, and differences between groups were evaluated by chi‐square analysis.

The general linear model or generalized linear model was utilized to compare the differences in BPI, WOMAC subtests, MMSE, and WMS‐CR subtest scores between two groups while controlling for age, gender, and years of education as covariates. For amygdala subregion volumes, age, gender, years of education, and whole amygdala volume were used as covariates with false discovery rate (FDR) applied to correct multiple comparisons.

We used partial correlation analysis to investigate the relationship between variables. In both KOA and HC groups, we controlled for age, gender, years of education, and whole amygdala volume as covariates when examining the association between volumes of amygdala subregions and cognitive function assessment scales. Within the KOA group, we adjusted for age, gender, and years of education while exploring the link between BPI scores and cognitive function assessment scales. Additionally, within the KOA group, we considered age, gender, years of education, and whole amygdala volume as covariates while investigating the association between volumes of amygdala subregions and PD‐1 levels.

## 4 RESULTS

### 4.1 Characteristics of demographic and clinical variables

The demographic and clinical variables of the KOA and HC groups are presented in Table S1. No significant differences were observed between the groups in terms of demographic variables, including age, gender, years of education, and BMI (*p *> .05).

### 4.2 Comparisons of clinical and cognitive assessments

Compared to the HC group, the KOA group exhibited significantly higher scores in BPI and WOMAC pain (standardized *β *= −.785; *p *< .001), WOMAC stiffness (standardized *β *= −.692; *p *< .001), and WOMAC Physical function (standardized *β *= −.827; *p *< .001). These findings indicate that individuals with KOA experienced more severe pain and had poorer knee function compared to the control group (Table S2).

Additionally, the KOA group demonstrated impaired cognitive performance in MMSE (standardized *β *= .591; *p *< .001), WMS‐CR mental control C (standardized *β *= .265; *p *= .032), WMS‐CR visual reproduction (standardized *β *= .378; *p *= .001), and WMS‐CR digit span (standardized *β *= .423; *p *< .001). This suggests a decline in global cognition, long‐delay free recall, short‐delay free recall, and immediate recall among individuals with KOA compared to the control group (Table 1). The results were obtained after controlling for covariates. The statistical results without covariates can be found in Tables S3 and S4.

**TABLE 1:** Comparisons of cognitive functions between the knee osteoarthritis group (KOA) and healthy control group (HC) groups.

	KOA (*n* = 36)	HC (*n* = 25)	Standardized *β*	*p*
**MMSE^a^**	28.00 (27.00–29.00)	30.00 (29.00–30.00)	.591	<.001
**WMS‐CR**				
Mental control A^a^	10.00 (9.00–12.00)	10.00 (7.50–11.00)	−.051	.677
Mental control B^a^	11.00 (10.00–12.00)	12.00 (10.00–12.00)	.096	.426
Mental control C^a^	10.50 (9.00–12.00)	12.00 (10.50–12.00)	.265	.032
Total mental control^a^	30.50 (27.00–34.00)	32.00 (28.50–34.00)	.133	.263
Picture recall^b^	9.97 ± 2.09	9.44 ± 2.40	−.093	.486
Visual recognition^a^	10.00 (9.00–12.00)	10.00 (8.00–12.00)	−.054	.677
Visual reproduction^a^	8.50 (7.00–10.75)	10.00 (9.00–12.00)	.378	.001
Associative learning^b^	9.19 ± 3.73	8.28 ± 3.36	−.131	.335
Touch^a^	10.00 (8.00–12.00)	9.00 (7.00–10.00)	−.207	.070
Comprehension memory^a^	7.00 (6.00–9.00)	6.00 (6.00–7.00)	−.149	.217
Digit span^a^	8.00 (7.00–10.00)	12.00 (10.00–15.00)	.423	<.001
Overall memory quotient (MQ)^b^	105.92 ± 15.23	110.56 ± 8.46	.143	.231

Abbreviations: MMSE: themini‐mentalstateexamination; WMS‐CR: the Wechsler Memory Scale‐Chinese Revision.

^a^Median (25th–75th percentile) was used for statistical description, and a generalized linear model was carried out with age, gender, and years of education as covariates.

^b^Mean ± SD was used for statistical description, and a general linear model was carried out with age, gender, and years of education as covariates.

### 4.3 Comparisons of amygdala subfield volumes

As shown in Table 2, after multiple comparison correction, the KOA group had significantly decreased amygdala subregion volumes of the lateral nucleus (standardized *β *= .107; the uncorrected *p* value [*p*_uncorr_] = .016; the corrected *p* value [*p*_FDR_] = .036), basal nucleus (standardized *β *= .059; *P*_uncorr _= .023; *P*_FDR_ **= .036), and paralaminar nucleus (standardized *β *= .137; *p*_uncorr _= .030; *p*_FDR_ **= .039), and increased amygdala subregion volumes of the accessory basal nucleus (standardized *β *= −.122; *p*_uncorr _= .008; *p*_FDR_ **= .030), central nucleus (standardized *β *= −.377; *p*_uncorr _= .001; *p*_FDR_ **= .009), medial nucleus (standardized *β *= −.283; *p*_uncorr _= .010; *p*_FDR_ **= .030), and cortical nucleus (standardized *β *= −.204; *p*_uncorr _= .024; *p*_FDR_ **= .036) compared with the HC group. The statistical results without covariates are shown in Table S5.

**TABLE 2:** Comparisons of amygdala subfield volumes between the knee osteoarthritis (KOA) and healthy control (HC) groups.

	KOA (*n* = 36)	HC (*n* = 25)	Standardized *β*	*p*_uncorr_	*p*_FDR_
**Lateral nucleus^a^ (mm^3^)**	1214.43 (1123.66–1277.77)	1266.42 (1184.77–1305.37)	.107	.016	.036
**Basal nucleus^a^ (mm^3^)**	768.06 (717.20–822.64)	800.53 (755.81–840.42)	.059	.023	.036
**Accessory basal nucleus^a^ (mm^3^)**	490.45 (427.74–522.46)	482.33 (457.89–504.12)	−.122	.008	.030
**Anterior amygdaloid area^b^ (mm^3^)**	84.08 ± 11.60	89.77 ± 12.36	.136	.079	.089
**Central nucleus^a^ (mm^3^)**	93.90 (81.47–103.23)	81.81 (78.29–92.49)	−.377	.001	.009
**Medial nucleus^b^ (mm^3^)**	46.87 ± 12.46	41.66 ± 9.48	−.283	.010	.030
**Cortical nucleus^b^ (mm^3^)**	51.11 ± 9.50	48.64 ± 7.20	−.204	.024	.036
**Cortico‐amygdaloid transition area^a^ (mm^3^)**	308.11 (273.78–331.16)	309.90 (290.24–318.65)	−.061	.277	.277
**Paralaminar nucleus^b^ (mm^3^)**	87.97 ± 11.28	93.98 ± 8.94	.137	.030	.039

*Note*: The unit of volume was cubic millimeters (mm^3^) and the left and right amygdala subfields were merged to calculate the volumes. *P*_uncorr_ indicated that it had not been corrected by the false discovery rate (FDR), and *P*_FDR_ indicated that it had been corrected by FDR.

^a^ Median (25th–75th percentile) was used for statistical description, and a generalized linear model was carried out with age, gender, years of education, and the volume of the whole amygdala as covariates.

^b^ Mean ± SD was used for statistical description, and a general linear model was carried out with age, gender, years of education, and the whole amygdala volume as covariates.

### 4.4 Partial correlation analysis

**FIGURE 1:**
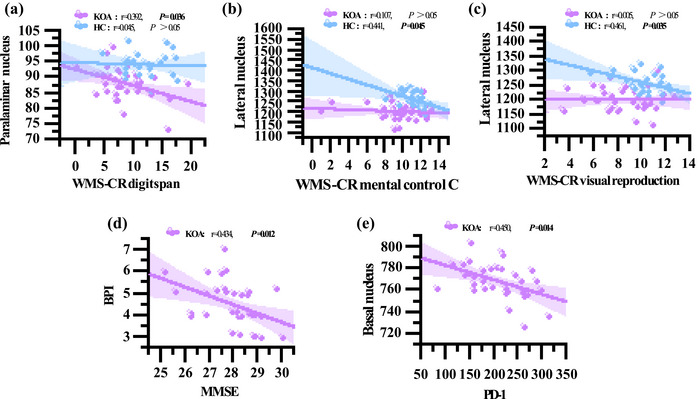
Partial correlation scatter plot. (a)–(c) Scatter plots indicate the association between amygdala subregion volumes and cognitive function scores in the knee osteoarthritis group (KOA) and healthy control group (HC) groups, with adjusted for age, sex, years of education, and the whole amygdala volume. (d) The scatter plot indicates the association between cognitive function scores and pain scores in the KOA group, with adjusted for age, gender, and years of education. (e) The scatter plot indicates the association between the amygdala subregion volumes and the levels of PD‐1 in the KOA group, with adjusted for age, sex, years of education, and the whole amygdala volume.

Table 3 presents partial correlation analysis results between amygdala subregion volumes and cognitive function scores in two groups, controlling for age, gender, years of education, and total amygdala volume. In the KOA group, a negative association was found between paralaminar nucleus volume and WMS‐CR digit span subtest scores (*r *= −.392; *p *= .036; Figure 1a). In the HC group, negative associations were observed between lateral nucleus volume and both WMS‐CR mental control *C* subtest scores (*r *= −.441; *p *= .045; Figure 1b) as well as WMS‐CR visual reproduction subtest scores (*r *= −.461; *p *= .035; Figure 1c). Additionally, controlling for age, gender, and years of education, there was a negative correlation between BPI scores and MMSE scores in the KOA group (*r *= −.434; *p *= .012; Table 4 ; Figure 1d).

**TABLE 3:** Partial correlation between amygdala subfield volumes and cognitive function scores in the knee osteoarthritis (KOA) and healthy control (HC) groups.

	Lateral nucleus		Basal nucleus		Accessory basal nucleus		Central nucleus		Medial nucleus		Cortical nucleus		Paralaminar nucleus	
	***r***	***p***	***r***	***p***	***r***	***p***	***r***	***p***	***r***	***p***	***r***	***p***	***r***	***p***
**KOA (*n* = 36)**														
**MMSE**	.187	.306	.142	.439	−.293	.104	−.084	.649	−.069	.706	−.235	.196	.216	.235
**WMS‐CR**														
Mental control C	−.107	.559	−.018	.921	.027	.883	−.072	.695	.034	.854	.036	.846	−.168	.357
Visual reproduction	−.005	.979	−.048	.793	.05	.787	−.005	.979	−.106	.564	.065	.723	−.106	.564
Digit span	−.043	.815	.028	.877	.083	.652	−.063	.731	−.094	.607	−.018	.923	−.392	.036
**HC (*n* = 25)**														
**MMSE**	−.092	.691	.036	.876	.109	.638	−.193	.403	−.194	.399	.025	.916	.246	.282
**WMS‐CR**														
Mental control C	−.441	.045	.099	.668	.367	.102	.300	.187	.273	.231	.230	.315	.094	.687
Visual reproduction	−.461	.035	.276	.225	.237	.300	.052	.822	.077	.741	.170	.462	.076	.743
Digit span	.112	.628	.069	.765	−.004	.986	−.329	.145	−.154	.506	−.046	.843	−.045	.845

*Note*: r: correlation coefficient; the unit of volume is cubic millimeters (mm^3^). Analysis was performed after adjusting for age, gender, years of education, and the whole amygdala volume.

Abbreviations: MMSE, themini‐mentalstateexamination; WMS‐CR, the Wechsler Memory Scale‐Chinese Revision.

**TABLE 4:** Partial correlation between cognitive function scores and pain scores in the knee osteoarthritis group (KOA) group.

	MMSE		WMS‐CR mental control C		WMS‐CR visual reproduction		WMS‐CR digit span	
	***r***	***p***	***r***	***p***	***r***	***p***	***r***	***p***
**BPI**	−.434	.012	−.101	.574	−.181	.312	−.290	.102

*Note*: *r*: correlation coefficient. Analysis was performed after adjusting for age, gender, and years of education.

Abbreviations: **BPI**, brief pain inventory; **MMSE**: themini‐mentalstateexamination; WMS‐CR, the Wechsler Memory Scale‐Chinese Revision.

**TABLE 5:** Partial correlation between the amygdala subfield volumes and the levels of programmed cell death protein‐1 (PD‐1) in the knee osteoarthritis group (KOA) group.

	Lateral nucleus		Basal nucleus		Accessory basal nucleus		Central nucleus		Medial nucleus		Cortical nucleus		Paralaminar nucleus	
	*r*	*p*	*r*	*p*	*r*	*p*	*r*	*p*	*r*	*p*	*r*	*p*	*r*	*p*
**PD‐1**	.086	.656	−.450	.014	.128	.510	.103	.596	.282	.138	.231	.228	−.186	.334

*Note*: The unit of volume is cubic millimeters (mm^3^). Analysis was performed after adjusting for age, gender, years of education, and the whole amygdala volume.

After controlling for age, gender, years of education, and the whole amygdala volume, the results of partial correlation analysis revealed a significant inverse association between the volume of basal nucleus in amygdala subfields and serum PD‐1 levels in the KOA group (*r *= −.450; *p *= .014; (Table 5). Figure 1e).

## 5 DISCUSSION

In this study, we conducted a comparative analysis of cognitive function and volumes of amygdala subregions between the KOA and HC groups, as well as their associations. Additionally, we explored the relationships between pain and cognitive function, as well as between amygdala subregion volumes and peripheral serum PD‐1 levels in the KOA group. Our findings revealed that compared to the HC group, the KOA group exhibited significant reductions in global cognition, long‐delay free recall, short‐delay free recall, and immediate recall. Moreover, inconsistent trends were observed in alterations of amygdala subregion volumes when compared with the HC group. Interestingly, a negative association was found between decreased volume of paralaminar nucleus and scores on WMS‐CR digit span test in the KOA group; BPI scores were negatively associated with MMSE scores; and decreased volume of basal nucleus was negatively associated with PD‐1 levels in the KOA group.

Consistent with previous research, individuals with KOA exhibited significant impairments in global cognition, long‐, and short‐delay free recall (Lin, G et al., 2022; Morone et al., 2014). Furthermore, a negative correlation was found between pain scores and global cognition, indicating that more severe pain symptoms are associated with poorer cognitive performance in KOA patients. Chronic pain induces neuroinflammation in brain regions such as the amygdala (Barcelon et al., 2019) and is positively correlated with inflammatory cytokine levels (Palada et al., 2019), leading to an increased activation of inflammatory factors in brain microglia and central nervous system inflammation (Inoue & Tsuda, 2018), resulting in synaptic remodeling and altered brain network function (McCarberg & Peppin, 2019). The presence of painful symptoms may affect cognitive function by increasing neuroinflammation within regions involved in cognitive processing among KOA patients. Additionally, our findings revealed decreased immediate recall ability among these individuals; this type of memory requires attentional resources for successful encoding (Botta et al., 2019). Patients experiencing chronic pain often focus excessively on their discomfort, which can lead to poor task performance due to reduced attentional capacity (van der Leeuw et al., 2018). We speculate that the presence of painful symptoms among KOA patients interferes with their ability to concentrate on tasks requiring immediate recall.

We observed a significant reduction in the volumes of the lateral, basal, and paralaminar subnuclei of the amygdala in individuals with KOA compared to HC group. Our findings are partially supported by a recent study that reported lower volumes of the basal and paralaminar nuclei in patients with chronic trigeminal neuralgia compared to healthy controls (Alper et al., 2021). The MMP‐9, a proteolytic zinc‐dependent enzyme, is the primary inflammatory mediator responsible for the induction of cartilage damage in KOA (Jayaram et al., 2023; Li et al., 2012). In the central nervous system, the involvement of MMP‐9 in neuroplasticity processes is pivotal, as it facilitates crucial aspects, such as neurogenesis, synaptogenesis, and myelination (Grochecki et al., 2023). It plays a significant role in synaptic plasticity and neurogenesis in brain regions such as the amygdala subregions like the basal nucleus (Gorkiewicz et al., 2015). We hypothesize that dysregulation of MMP‐9 levels in the KOA population may be a potential factor leading to changes in amygdala subregion volumes. However, additional studies are necessary to confirm this hypothesis.

In this study, we identified augmented volumes of the accessory basal nucleus, central nucleus, medial nucleus, and cortical nucleus in individuals with KOA. The accessory basal nucleus functions as a principal input pathway to the central amygdala (Fudge & Tucker, 2009). Within the amygdala complex, the central nuclei play a pivotal role in processing chronic pain signals (Lin, Y. L. et al., 2022). Although serving as an essential route for pain relief mechanisms (O'Neill & Meszaros, 2020), the connection linking the central nuclei with the parabrachial complex; on another note, the link connecting amygdaloid's central nucleus with the anterior nucleus of paraventricular thalamus contributes to pain sensitization processes (Chang et al., 2019). Furthermore, both medial and cortical nuclei significantly contribute to modulating persistent nociceptive stimuli (Werka, 1997). Based on our observations, we postulate that these enlarged volumes of specific subregions within the amygdala might signify a compensatory mechanism associated with chronic pain among individuals diagnosed with KOA.

Interestingly, our findings reveal a negative correlation between reduced volume of the paralaminar nucleus and immediate recall in the KOA group, thereby confirming previous research that suggests an association between diminished amygdala subregion volumes and decline in immediate recall (Liao et al., 2021). The paralaminar nucleus encompasses an immature subgroup of neurons that merge into the cortico‐amygdaloid transition area, which is also where the hippocampal CA1 area projects to (Fudge et al., 2012). Immediate memory has been linked to CA1 volume (La et al., 2019), implying that this may elucidate the observed correlation between paralaminar nucleus volume and immediate recall in the KOA group.

The correlation between basal nucleus volume and peripheral serum PD‐1 levels in KOA patients is significant. PD‐1, an immunotherapeutic target, binds to its ligand PD‐L1 to suppress T‐cell inflammatory activity (Zhao et al., 2021) and inhibit pain neuron function (Chen et al., 2017), which plays a crucial role in pain regulation and disease progression in individuals with KOA (Liu, S et al., 2019; Montesino‐Goicolea et al., 2022). However, decreased PD‐1 levels may have a protective effect on cognitive function (Zhao J et al., 2021), as studies have shown that blocking the PD‐1/PD‐L1 pathway can reduce amyloid‐beta plaque deposition and enhance cognitive function in mouse models of Alzheimer's disease (Baruch et al., 2016; Rosenzweig et al., 2019). Additionally, elevated peripheral serum levels of both PD‐1 and PD‐L1 have been observed in patients with Alzheimer's disease compared to healthy individuals (Wu et al., 2022). Previous research has also linked reduced basal nucleus volume with impaired cognitive performance (Liu et al., 2022), whereas increased peripheral serum levels of PD‐1 were found in KOA patients compared to healthy controls (Shan et al., 2017). Therefore, we hypothesize that the relationship between cognitive deficits and amygdala subregion volumes may be mediated by the levels of PD‐1 present in individuals with KOA.

Our study had several limitations. First, the sample size was limited. However, we controlled for age, gender, years of education, and whole amygdala volume as covariates when comparing differences between the two groups. Despite applying multiple comparison correction methods, significant differences in volumes of amygdala subregions were still observed, indicating robust findings. Additionally, it is worth considering that the potential gender disparity among participants in this specific study may have influenced the outcomes, as previous research has indicated that gender can also impact amygdala volume (DeCasien et al., 2022) and serum PD‐1 levels (Gu et al., 2022). Previous studies have reported a higher incidence of KOA in women than in men, which is consistent with our current study (Novin et al., 2023). The inclusion of a larger number of male KOA subjects in future studies is recommended to enhance the validity and robustness of the findings presented in this study. Third, blood samples were not collected from HC group; thus, a direct comparison of peripheral serum PD‐1 levels between KOA patients and HC individuals could not be conducted. However, Shan et al.’s study demonstrated increased peripheral serum PD‐1 levels in KOA individuals compared to healthy controls (Shan et al., 2017). Although our study suggests a potential association between cognitive decline and PD‐1 levels in the peripheral blood of KOA individuals, larger studies are warranted to confirm this hypothesis. Finally, due to the cross‐sectional design employed in our study, causality cannot be established; therefore, longitudinal studies are necessary to determine whether changes in volumes of amygdala subregions over time are associated with cognitive decline and peripheral serum PD‐1 levels.

## 6 CONCLUSION

Our findings suggest that individuals with KOA exhibit significant impairments in global cognition and memory function compared to HC group. Moreover, the severity of pain experienced by KOA patients is significantly associated with the degree of cognitive impairment observed. Additionally, our results indicate abnormal alterations in subregions of the amygdala among KOA patients when compared to HC group. These changes in amygdala subregion volumes are significantly linked to immediate recall ability and serum PD‐1 levels, highlighting a potential mechanism underlying cognitive decline in KOA.

## AUTHOR CONTRIBUTIONS

**Peiling Zeng**: Investigation; formal analysis; writing—original draft; visualization. **Baoru Zhao**: Investigation; formal analysis; writing—original draft; visualization. **Ming Li**: Investigation; visualization; writing—original draft; formal analysis. **Yajun Wang**: Resources; data curation; writing—review and editing. **Guiyan Cai**: Data curation; resources; writing—review and editing. **Ruilin Chen**: Writing—review and editing; data curation; resources. **Lidian Chen**: Conceptualization; methodology; writing—review and editing; supervision; funding acquisition. **Jiao Liu**: Conceptualization; methodology; funding acquisition; writing—review and editing; supervision.

## CONFLICT OF INTEREST STATEMENTS

The authors declare no conflicts of interest.

### PEER REVIEW

The peer review history for this article is available at https://publons.com/publon/10.1002/brb3.70042.

## TRIAL REGISTRATION

Chinese Clinical Trial Registry (ChiCTR‐IOR‐16009308)

## Supporting information



Supporting Information

## Data Availability

Data from this study are available from the corresponding author upon request, where reasonable.
