# Supplementary material for: The volumes of amygdala subregions and peripheral programmed cell death protein‐1 levels are associated with cognitive decline in individuals with knee osteoarthritis
Source: Brain Behav. 2024 Sep 30;14(10):e70042. doi: 10.1002/brb3.70042 (PMC11633366; doi:10.1002/brb3.70042)
Supplement: Supplementary file 1 — Supporting Information [file BRB3-14-e70042-s001.docx]

**Table S1. Demographics characteristics of participants.**

|  | **KOA(n=36)** | **HC(n=25)** | ***t/Z/χ^2^*** | ***P*** |
| --- | --- | --- | --- | --- |
| **Age (years) ^a^** | 60.00(59.00~66.75) | 62.00(57.50~68.00) | -0.757 | 0.449 |
| **Gender [n (%)]** |  |  |  |  |
| **Male[*n* (%)] ^b^** | 8(22.20) | 8(32.00) | 0.729 | 0.393 |
| **Female [*n* (%)] ^b^** | 28(77.80) | 17(68.00) |  |  |
| **Education(years) ^c^** | 11.28±3.90 | 11.32±2.97 | -0.046 | 0.964 |
| **BMI ^c^** | 22.98±2.72 | 23.41±2.56 | -0.620 | 0.537 |

**KOA**: Knee Osteoarthritis group; **HC**: Healthy Control group; **BMI**: Body Mass Index.^a^ Mann–Whitney U test was adopted, and median (25~75th percentile) was used for statistical description; ^b^ *chi-square* test was adopted, and n(%) was used for statistical description; ^c^ two independent sample t-test was adopted, and mean±SD was used for statistical description.

**Table S2. Comparisons of the BPI score and WOMAC sub-scores of the KOA and HC groups.**

|  | **KOA(n=36)** | **HC(n=25)** | **Standardized *β*** | ***P*** |
| --- | --- | --- | --- | --- |
| **BPI ^a^** | 4.00(4.00~5.00) | 0.00(0.00~0.00) | - | - |
| **WOMAC pain ^ab^** | 4.00(3.00~6.75) | 0.00(0.00~0.00) | -0.785 | ＜0.001 |
| **WOMAC stiffness ^ab^** | 3.00(1.25~4.00) | 0.00(0.00~0.00) | -0.692 | ＜0.001 |
| **WOMAC Physical function ^ab^** | 13.50(9.25~17.00) | 0.00(0.00~0.00) | -0.827 | ＜0.001 |

**KOA**: Knee Osteoarthritis group; **HC**: Healthy Controls group; **BPI**: Brief Pain Inventory; **WOMAC**: the Western Ontario and McMaster Universities Arthritis Index; **WOMAC pain**: the WOWAC subtest of pain; **WOMAC stiffness**: the WOWAC subtest of stiffness; **WOMAC Physical function**: the WOWAC subtest of Physical function. ^a^ Median (25~75th percentile) was used for statistical description; ^b^ A generalized linear model was carried out with age, gender and years of education as covariates.

**Table S3. Comparisons of the BPI score and WOMAC sub-scores of the KOA and HC groups without covariates.**

|  | **KOA(n=36)** | **HC(n=25)** | ***Z*** | ***P*** |
| --- | --- | --- | --- | --- |
| **BPI ^a^** | 4.00(4.00~5.00) | 0.00(0.00~0.00) | - | - |
| **WOMAC pain ^a^** | 4.00(3.00~6.75) | 0.00(0.00~0.00) | -6.746 | ＜0.001 |
| **WOMAC stiffness ^a^** | 3.00(1.25~4.00) | 0.00(0.00~0.00) | -5.777 | ＜0.001 |
| **WOMAC Physical function ^a^** | 13.50(9.25~17.00) | 0.00(0.00~0.00) | -6.721 | ＜0.001 |

**KOA**: Knee Osteoarthritis group; **HC**: Healthy Controls group; **BPI**: Brief Pain Inventory; **WOMAC**: the Western Ontario and McMaster Universities Arthritis Index; **WOMAC pain**: the WOWAC subtest of pain; **WOMAC stiffness**: the WOWAC subtest of stiffness; **WOMAC Physical function**: the WOWAC subtest of Physical function. ^a^ Median (25~75th percentile) was used for statistical description, and the *Mann-Whitney U test* was used for analysis.

**Table S4. Comparisons of cognitive functions between the KOA and HC groups without covariates.**

|  | **KOA(n=36)** | **HC(n=25)** | ***Z/t*** | ***P*** |
| --- | --- | --- | --- | --- |
| **MMSE ^a^** | 28.00(27.00~29.00) | 30.00(29.00~30.00) | -4.841 | <0.001 |
| **WMS-CR** |  |  |  |  |
| Mental control A ^a^ | 10.00(9.00~12.00) | 10.00(7.50~11.00) | -1.274 | 0.203 |
| Mental control B ^a^ | 11.00(10.00~12.00) | 12.00(10.00~12.00) | -0.942 | 0.346 |
| Mental control C ^a^ | 10.50(9.00~12.00) | 12.00(10.50~12.00) | -1.888 | 0.059 |
| Total mental control ^a^ | 30.50(27.00~34.00) | 32.00(28.50~34.00) | -0.486 | 0.627 |
| Picture recall ^b^ | 9.97±2.09 | 9.44±2.40 | 0.920 | 0.361 |
| Visual recognition ^a^ | 10.00(9.00~12.00) | 10.00(8.00~12.00) | -0.396 | 0.692 |
| Visual reproduction ^a^ | 8.50(7.00~10.75) | 10.00(9.00~12.00) | -3.066 | 0.002 |
| Associative learning ^b^ | 9.19±3.73 | 8.28±3.36 | 0.980 | 0.331 |
| Touch ^a^ | 10.00(8.00~12.00) | 9.00(7.00~10.00) | -1.763 | 0.078 |
| Comprehension memory ^a^ | 7.00(6.00~9.00) | 6.00(6.00~7.00) | -1.824 | 0.068 |
| Digit span ^a^ | 8.00(7.00~10.00) | 12.00(10.00~15.00) | -3.653 | ＜0.001 |
| Overall memory quotient (MQ) ^b^ | 105.92±15.23 | 110.56±8.46 | -1.381 | 0.172 |

**KOA**: Knee Osteoarthritis group; **HC**: Healthy Control group; **MMSE**: [the Mini-Mental State Examination](https://pubmed.ncbi.nlm.nih.gov/34607584/); **WMS-CR**: the Wechsler Memory Scale-Chinese Revision. ^a^ Median (25~75th percentile) was used for statistical description, and the *Mann-Whitney U test* was used for analysis; ^b^ Mean ± SD was used for statistical description, and the two independent sample *t test* was used for analysis.

**Table S5. Comparisons of** **amygdala subfield volumes between the KOA and HC groups without covariates.**

|  | **KOA(n=36)** | **HC(n=25)** | ***Z/t*** | ***P*** |
| --- | --- | --- | --- | --- |
| **Lateral nucleus ^a^** | 1214.43(1123.66~1277.77) | 1266.42(1184.77~1305.37) | -2.112 | 0.035 |
| **Basal nucleus ^a^** | 768.06(717.20~822.64) | 800.53(755.81~840.42) | -1.628 | 0.104 |
| **Accessory Basal nucleus ^a^** | 490.45(427.74~522.46) | 482.33(457.89~504.12) | -0.293 | 0.769 |
| **Anterior amygdaloid area ^b^** | 84.08±11.60 | 89.77±12.36 | -1.835 | 0.072 |
| **Central nucleus ^a^** | 93.90(81.47~103.23) | 81.81(78.29~92.49) | -2.156 | 0.031 |
| **Medial nucleus ^b^** | 46.87±12.46 | 41.66±9.48 | 1.766 | 0.083 |
| **Cortical nucleus ^b^** | 51.11±9.50 | 48.64±7.20 | 1.101 | 0.275 |
| **Cortico-amygdaloid transition area ^a^** | 308.11(273.78~331.16) | 309.90(290.24~318.65) | -0.425 | 0.671 |
| **Paralaminar nucleus ^b^** | 87.97±11.28 | 93.98±8.94 | -2.219 | 0.030 |

**KOA**: Knee Osteoarthritis group; **HC**: Healthy Control group; The unit of volume was cubic millimeters and the left and right amygdala subfields were merged to calculate the volumes. ^a^ Median (25~75th percentile) was used for statistical description, and the *Mann-Whitney U* *test* was used for analysis;^b^ Mean ± SD was used for statistical description, and the two independent sample *t test* was used for analysis.
